# Opportunities for Regulatory Changes to Promote Pediatric Device Innovation in the United States: Joint Recommendations From Pediatric Innovator Roundtables

**DOI:** 10.1109/JTEHM.2021.3092559

**Published:** 2021-06-25

**Authors:** Terence Sanger, Anthony Chang, William Feaster, Sharief Taraman, Nadine Afari, Debra Beauregard, Brent Dethlefs, Tiffani Ghere, Mustafa Kabeer, George Tolomiczenko, Michael Billig, Jon Brophy, Kolaleh Eskandanian, Juan Espinoza, Sherry Farrugia, Michael Harrison, Christopher Horvat, Claudia Hoyen, Chester Koh, Allison Komiyama, Krista Nelson, Omkar Kulkarni, Robert Levy, Kevin Maher, Michael O’Donnell, Todd Ponsky, Frances Richmond, Jessica Richter, Shuvo Roy, Shreim Samir, Srinivasan Suresh, Charlette Stallworth, Usha Thekkedath, Kara Toman, James Wall, Leanne West, Dawn Wolff

**Affiliations:** 1 Children’s Hospital of Orange County (CHOC)OrangeCA92868USA; 2 University of California Irvine 8788 IrvineCA92697USA; 3 CognoaPalo AltoCA94306USA; 4 Caltech 6469 PasadenaCA91125USA; 5 Experien GroupSan DiegoCA92127USA; 6 Cincinnati Children’s Hospital Medical Center 2518 CincinnatiOH45229USA; 7 Children’s National HospitalWashingtonDC20010USA; 8 Children’s Hospital of Los Angeles 5150 Los AngelesCA90027USA; 9 West Coast Consortium for Technology & Innovation in PediatricsLos AngelesCA94612USA; 10 Global Center for Medical InnovationAtlantaGA30318USA; 11 University of California at San Francisco 8785 San FranciscoCA94143USA; 12 UCSF-Stanford PDCSan FranciscoCA94158USA; 13 University of Pittsburgh 6614 PittsburghPA15260USA; 14 UPMC Children’s Hospital of PittsburghPittsburghPA15224USA; 15 University Hospitals Rainbow Babies and Children’s HospitalClevelandOH44106USA; 16 Case Western Reserve University School of Medicine 12304 ClevelandOH44106USA; 17 Texas Children’s Hospital 3984 HoustonTX77030USA; 18 Southwest National Pediatric Device ConsortiumHoustonTX77030USA; 19 Acknowledge Regulatory StrategiesSan DiegoCA92110USA; 20 Children’s Mercy Hospital 4204 Kansas CityMO64108USA; 21 Children’s Hospital of Philadelphia 6567 PhiladelphiaPA19104USA; 22 Children’s Healthcare of AtlantaAtlantaGA30329USA; 23 University of California at Berkeley 1438 BerkeleyCA94720USA; 24 International Center for Regulatory ScienceUniversity of Southern California 5116 Los AngelesCA90007USA; 25 Cactus MedicalIrvineCA92612USA; 26 Stanford Children’s HealthLucile Packard Children’s Hospital 24349 Palo AltoCA94304USA; 27 Georgia Tech Research Institute (GTRI)AtlantaGA30318USA

**Keywords:** Device, FDA, guidelines, pediatric, regulatory

## Abstract

Objective: The purpose of this report is to provide insight from pediatric stakeholders with a shared desire to facilitate a revision of the current United States regulatory pathways for the development of pediatric healthcare devices. Methods: On August 5, 2020, a group of innovators, engineers, professors and clinicians met to discuss challenges and opportunities for the development of new medical devices for pediatric health and the importance of creating a regulatory environment that encourages and accelerates the research and development of such devices. On January 6, 2021, this group joined regulatory experts at a follow-up meeting. Results: One of the primary issues identified was the need to present decision-makers with opportunities that change the return-on-investment balance between adult and pediatric devices to promote investment in pediatric devices. Discussion/Conclusion: Several proposed strategies were discussed, and these strategies can be divided into two broad categories: 1. Removal of real and perceived barriers to pediatric device innovation; 2. Increasing incentives for pediatric device innovation.

## I. Introduction

On August 5, 2020, a group of innovators, engineers, and clinicians met to discuss challenges and opportunities for the development of new devices for pediatric health and the importance of creating a regulatory environment that encourages the development of such devices. This group, which comprises experts affiliated with academic institutions, hospitals, and industry (Table 1), strongly desires to see the landscape changed to support investments in pediatric device development and to create a strong value proposition illustrating the opportunities within this market.

**TABLE 1:** Professional Affiliation for Participants in the Pediatric Innovator Roundtables

	Number	%
Affiliation^a^		
Academia	13	–
Hospital	30	–
Industry	9	–
Profession		
Medicine		
Cardiology	3	8.1
Critical Care	1	2.7
General	4	10.8
Infectious Diseases	1	2.7
Neurology	2	5.4
Surgery	5	13.5
Urology	1	2.7
Other	20	54.1

^a^ Some stakeholders were affiliated with numerous institutions.

On January 6, 2021, this group joined regulatory experts at a follow-up meeting to discuss challenges and opportunities for change in the regulatory environment that will have a meaningful and significant impact on efforts to develop pediatric medical devices, which the Federal Food, Drug, and Cosmetic Act defined as devices used to treat or diagnose diseases and conditions from birth through 21 years of age [1].

Some pediatric medical devices are designed specifically for children, while others are derived from products designed for adults. The challenges associated with designing medical devices for use in pediatric patients include smaller body size and higher activity level compared with adults, changes in body structures and functions throughout childhood, and the fact that pediatric devices may be used over the long term, necessitating additional research on device longevity and long-term exposure to implanted materials [1].

During the period from 2008 to 2018, only 10% of pre-market approvals and humanitarian device exemptions (HDEs) granted by the U.S. Food and Drug Administration (FDA) were awarded for devices with indications for use in patients <18 years of age. Only 4% were for devices indicated for use in patients 0–2 years of age. Only four devices approved for HDEs were designed specifically for the pediatric population [2].

The challenges of developing devices specifically for pediatric patients are also market driven. One of the primary issues identified was the need to present decision-makers, whether early-stage investors in a start-up or executives at a large device company, with opportunities that change the return-on-investment (ROI) balance between adult and pediatric devices to promote investment in the latter. Measures are urgently needed to offset the costs of testing, manufacturing, marketing, and distributing new pediatric medical devices. At the FDA Pediatric Medical Device Development Public Meeting (2018), 76% of participants in an audience poll reported aspects of ROI as the dominant barriers to entering the pediatric medical device market [2]. Our roundtable discussions of these challenges led to proposal of the following strategies, which may be divided into two broad categories:

1. Removal of real and perceived barriers to innovation
2. Increasing incentives for pediatric device innovation.

## II. Review

Below, we review the results of our qualitative study of the opportunities for regulatory changes to promote pediatric device innovation in the United States. The solutions to current challenges will be multifactorial in nature. Roundtable participants were unable to identify the most important barriers as such due to a lack of data prioritization and/or ranking. The concepts presented below are not prioritized in a particular order and will need to be considered concurrently.

### A. Removal of Real and Perceived Barriers to Pediatric Device Innovation

One of the primary barriers to the innovation of pediatric devices is the perception that investigational research in pediatric populations carries greater legal, ethical, and public relations risks than similar research in adults. While there are indeed additional protections for pediatric patients as a vulnerable population, it is important to recognize that excessive limitations for the testing and validation of pediatric medical devices may result in the denial of important medical advances [3]. In the collective experience of the meeting group, the rigorous standards applied to adult devices are often adequate for pediatric medical devices. Further investigation into this topic, including a review of the impacts of pediatric adverse events, will be required to support detailed recommendations. An accurate assessment of relative risk in pediatric vs. adult populations will contribute to the establishment of guidelines for ethical research in pediatrics. Such guidelines can provide a more uniform and standardized review process for institutional review boards (IRBs), the FDA, the National Institutes of Health (NIH), and hospital ethics panels. New guidelines may be particularly important for the legal protection of personnel in small companies, for whom personal legal or financial liability for adverse events may be a strong personal disincentive for participation in pediatric device innovation. Revised guidance documents will be most effective when they are relevant, succinct, and developed on a rapid timeline.

### B. Increase Education on the Process of Pediatric Device Development

Another barrier to pediatric device innovation is inconsistency in levels of pediatric-related knowledge among startups, companies, regulatory experts, ethics review panels, and government reviewers. Despite the existence of FDA guidelines for pediatric devices, some of the personnel involved in the review process are not as familiar with these regulatory guidance documents as they are with those for adult devices. This can lead to excessive caution and create unnecessary delays in the development of pediatric devices of significant benefit. Overcoming this barrier requires improved education on pediatric devices for all stakeholders, including regulatory personnel, innovators, and corporate leadership. Augmented learning models will allow inventors and device developers to better understand complex issues, to acquire relevant clinical and engineering information at the right time during the R&D process, and to correctly assess the technical risks that can delay testing, approval, and development. Further, the inclusion of pediatricians and pediatric subspecialists on regulatory review panels and advisory boards will ensure that the unique perspective and experience of pediatric clinicians is incorporated into innovation and regulatory approval processes. Such attention to the specific needs of pediatric patients may result in a more condition-focused, rather than device-focused, approach, thus maximizing impact and contributing to a more effective and appropriate FDA review process that respects the necessity of new pediatric devices for improved health outcomes.

Pediatric-specific educational programs for students and professional innovators will facilitate smooth progression through the regulatory process. Roundtable participants proposed programs that include government-supported stipends for individuals or teams to pursue early stages of innovation to advance a concept to the point where additional funding might be obtainable. The goal for this type of funding is to provide support for the innovator, rather than the product, with the expectation that doing so will lead to successful pediatric products. With the current lack of regulation to support pediatric device development, “research and development” have been split de facto, with research most heavily concentrated within universities, and design and development most often concentrated in privately held companies. A smoother transition to private funding and earlier regulatory approval will be supported by encouraging basic research within small and medium-sized companies, and by educating university students and faculty on methods pertinent to early phase design and development. Academic institutions are important partners with industry in this process because they are uniquely positioned to develop approaches to innovation and collaboration with the FDA and other government agencies. Academic institutions can work closely with industry and the FDA to develop pediatric-focused talent pipelines; students and faculty in these programs will bring together knowledge from a range of scientific disciplines to assess quality and safety and to inform regulatory decision-making throughout a device’s lifecycle. These programs would allow for three-way dialogue and facilitate cross-disciplinary collaboration.

The FDA’s Center for Devices and Radiological Health (CDRH) provides information to help industry comply with FDA regulations. CDRH works closely with the FDA Office of Orphan Products Development (OOPD) to evaluate scientific and clinical data submissions from sponsors to identify medical products with promise for the treatment of rare disease and to further advance the scientific development of such products.

The CDRH also developed the System of Hospitals for Innovation in Pediatrics–Medical Devices (SHIP-MD). SHIP-MD aims to improve public health for children through transformation of the pediatric medical device ecosystem by de-risking and accelerating product development to stimulate investment and innovation in pediatric devices. This effort was developed and guided by a multi-stakeholder group that included the Critical Path Institute (C-Path), the CDRH, AdvaMed, the American Academy of Pediatrics (AAP), and various leaders of pediatric health systems.

### C. Address Challenges Specific to Pediatric Research

Most medical devices used in pediatrics are never studied in the intended patient population, due to the small numbers of patients affected, the increased protections surrounding pediatric research, and the lack of financial incentive. The FDA’s Real World Evidence (RWE) program provides a new path to FDA clearance/approval for devices used in pediatric patients and other small populations for whom traditional randomized controlled trials are impractical by lowering costs and by removing hurdles to data collection [4]. RWE program analysis has demonstrated how healthcare systems collect, store, and curate data. While the goal of the RWE program is faster approval of medical devices intended for pediatric populations and indications, data sets need to be standardized to ensure data quality, safety, privacy, and reliability. The UCSF-Stanford Pediatric Device Consortia (PDC) are working with AtriCure, Inc. and recently demonstrated the use of RWE in supporting the regulatory clearance of new pain therapy devices (cryoablation nerve block) for adolescent use [5], [6]. The FDA’s acceptance of RWE has the potential to transform the regulatory landscape for pediatric devices. The RWE program can thus close evidence gaps by demonstrating the actual value of pediatric medical devices in patients under real-world conditions.

Because of the wide variety of disorders that present in childhood, many pediatric devices are designed for rare disorders. This poses difficulties for achieving adequate power in safety and efficacy trials. The symptoms and etiology of pediatric disease are often more heterogeneous than those of adult disease, in part because most adult disease is acquired on a background of prior health, whereas childhood disorders affect multiple aspects of development. Furthermore, the greatest effect of an intervention in childhood may not be seen for many years, and the resulting health impacts may not fully manifest until adulthood. Therefore, rigid adherence to prospective randomized trials in well-defined cohorts is problematic and often does not reflect the use of pediatric devices in actual clinical practice. Overcoming this barrier will require greater opportunity for creativity in the design of clinical trials, including delayed entry, intent-to-treat analysis, personalized outcome measures, and post-hoc subgroup analysis.

Effective research for pediatric device innovation will require greater ability to rely on RWE from post-market use of the proposed device or similar devices. Participants in the January 6, 2021 roundtable strongly encouraged the creation of pediatric-specific guidelines for clinical trials and the establishment of a post-market registry for the uniform and comprehensive accumulation of real-world data, off-label uses, and post-market experience. Challenges related to the statistical analysis and curation of these data remain to be addressed. Efforts to obtain corporate data for the registry will be aided by regulations that reduce the legal and regulatory risks incurred by corporations that report negative as well as positive outcomes in good faith. In this context, the FDA may function as a neutral party to obtain reports of off-label pediatric treatments in support of expanded labeling. A concept called “reverse extrapolation” would also harness the use of data collected during pediatric medical device studies to support the approval of adult indications and thus accelerate device development [7].

### D. Mitigate Logistical Barriers

Finally, measures may be taken to address the barriers to market entry that deter small companies seeking to innovate pediatric devices. Such barriers include the increasing complexity of animal testing requirements, file formats for application submission, and documentation required for device development, manufacturing, and testing.

## III. Discussion

### A. Regulations That Enforce the Development of Pediatric Versions of Adult Devices

A corporation that is considering investing in the development of an adult device has very little incentive to produce a pediatric version of that device. Although there is potential for market expansion, the need for pediatric-specific safety and efficacy testing, the need to manufacture, stock, and distribute devices in multiple sizes, and the perceived legal and ethical risks act as strong deterrents. This barrier could be partly overcome by a mechanism similar to the requirement that pharmaceuticals brought to market must undergo pediatric testing and labeling. However, the use of regulation in this manner may provide unnecessary burdens on smaller pharmaceutical companies, and it would favor investment in adult devices for which no pediatric equivalent would be useful or possible.

Furthermore, the pediatric patient population encompasses a wide range of development, from 0 to 21 years of age [1]. The policies proposed here could incentivize efforts to develop a device that is suitable for a subset of this population, such as adolescents (age 12–21 years), without the need to commit to ensuring device efficacy across the full range of pediatric needs, from neonates to 21-year-olds.

### B. Incentives for Devices With Dual Use in Adult and Pediatric Populations

For this reason, the participants strongly suggest the use of incentives, with or without enforcement regulations. Several incentives already exist, including a reduced or zero cost for FDA submission, the FDA’s breakthrough devices program with Medicaid reimbursement incentives, as well as the upcoming safer technologies program [8]. Additional incentives could be modeled on the pediatric priority review vouchers program for pharmaceutical development, as this has proven to be a valuable motivator for companies. Roundtable participants emphasized that incentives may need to be different for small, medium, and large-sized companies, and thus a range of potential options should be made available. Financial incentives could include reimbursement guarantees, extended patent protection, a vouchers program for pediatric devices, and accepting the results of pediatric clinical testing and post-market experience as a foundation for subsequent adult labeling. Non-financial incentives could include expedited FDA review panels, allowing requests for specific reviewers and recommendations for outside reviewers, additional guidance and support prior to submission, pre-review prior to formal regulatory submission, and expediting the review of resubmissions for devices intended for combined pediatric and adult labeling.

### C. Incentives That Decrease Risk and Increase Return on Investment

In a financially competitive environment, pediatric device development and labeling will only be funded when the ROI exceeds that for the development of adult devices. For example, the UCSF-Stanford Pediatric Device Consortia (PDC) were consulting a company interested in developing a pediatric version of their adult device. This company was ready to invest some effort and money in design modifications and limited clinical trials for regulatory submissions. However, on discussion with a variety of pediatricians at UCSF, they came to an understanding that the off-label use of adult devices is a common/accepted practice (they were even told that it is up to them if they want to invest in a “pediatric device” and get approvals). Hence, they were debating the need/incentive for the extra effort and related expenses.

The process of securing approval for pediatric devices will be straightforward for companies that have pediatric expertise and position themselves in this niche market. However, such companies must still compete for funds with the innovators of adult devices. While removing the barriers identified above will help to reduce the risks associated with pediatric device development, even if the perceived risk is reduced to match that of developing an equivalent adult device, the smaller pediatric market cannot match the ROI for adult products. Therefore, if companies seeking to innovate pediatric devices are to compete successfully, the costs and risks for pediatric devices must be less than those for adult devices. Financial incentives could include reimbursement guarantees through both Medicaid and Medicare, extended patent protection, special grant programs through the NIH, incentivizing tax credits, and pediatric innovation or venture philanthropy funds. Notably, the FDA does not charge filing fees for some devices designated solely for pediatric use [9]. Roundtable participants believe that such measures should be continued and/or expanded.

Non-financial incentives could include:

• a fast-track approval process
• expedited and pediatric-specific FDA review panels
• allowing requests for specific reviewers and recommendations for outside reviewers with pediatric expertise
• increased staffing of pediatric device regulatory offices (including OOPD)
• additional pre-review guidance and support prior to regulatory submission
• expedited response to resubmissions
• support for programs to educate participants on the regulation of pediatric device development
• pediatric-specific guidelines for clinical trials
• the use of post-market as well as pre-market and post-market data for predicate devices approved for use in adults
• a pediatric-specific registry for real-world data.

Roundtable participants encouraged a paradigm shift to seeing the FDA and industry as partners in the design and approval of high-impact pediatric devices. In addition to the regulatory changes listed above, a “pledge” or “pediatric innovation seal” could be developed for companies that meet criteria for innovation in pediatrics. Future efforts should include parents and patients in determining priorities and goals for pediatric health and in providing the advocacy support necessary to effect change. Larger companies might be willing to support philanthropy venture funds for pediatrics if the act led to recognition of their involvement as a “good neighbor” in the healthcare community.

## IV. Summary

Ultimately, there will be no single solution. The consensus outcome of this meeting is that there are multiple opportunities, and a flexible combination of new programs and regulatory changes can be implemented to benefit the multiple stakeholders in pediatric device development. An essential component will be building a cadre of experts with the development, regulatory, and clinical expertise to support all innovators. Ongoing work from groups including this working group, the FDA-SHIP program, the International Society for Pediatric Innovation (iSPI), the PDC, and other groups of experts will continue to contribute new ideas and opportunities for increasing the quantity, quality, and efficacy of pediatric device innovation, labeling, marketing, and implementation in clinical practice [5], [10], [11].
